# Communication and Control by Listening: Toward Optimal Design of a Two-Class Auditory Streaming Brain-Computer Interface

**DOI:** 10.3389/fnins.2012.00181

**Published:** 2012-12-19

**Authors:** N. Jeremy Hill, Aisha Moinuddin, Ann-Katrin Häuser, Stephan Kienzle, Gerwin Schalk

**Affiliations:** ^1^New York State Department of Health, Wadsworth CenterAlbany, NY, USA; ^2^Department of Biomedical Engineering, University of MichiganAnn Arbor, MI, USA; ^3^Institute for Cognitive Science, University of OsnabrückOsnabrück, Germany; ^4^Briarcliff High School, Briarcliff ManorNY, USA; ^5^Department of Neurology, Albany Medical CollegeAlbany, NY, USA; ^6^Department of Neurosurgery, Washington UniversitySt. Louis, MO, USA; ^7^Department of Biomedical Engineering, Rensselaer Polytechnic InstituteTroy, NY, USA; ^8^Department of Biomedical Sciences, State University of New YorkAlbany, NY, USA; ^9^Department of Electrical and Computer Engineering, University of TexasEl Paso, TX, USA

**Keywords:** brain-computer interface, auditory event-related potentials, N1 potential, P3 potential, dichotic listening, auditory attention

## Abstract

Most brain-computer interface (BCI) systems require users to modulate brain signals in response to visual stimuli. Thus, they may not be useful to people with limited vision, such as those with severe paralysis. One important approach for overcoming this issue is auditory streaming, an approach whereby a BCI system is driven by shifts of attention between two simultaneously presented auditory stimulus streams. Motivated by the long-term goal of translating such a system into a reliable, simple yes-no interface for clinical usage, we aim to answer two main questions. First, we asked which of two previously published variants provides superior performance: a fixed-phase (FP) design in which the streams have equal period and opposite phase, or a drifting-phase (DP) design where the periods are unequal. We found FP to be superior to DP (*p* = 0.002): average performance levels were 80 and 72% correct, respectively. We were also able to show, in a pilot with one subject, that auditory streaming can support *continuous* control and neurofeedback applications: by shifting attention between ongoing left and right auditory streams, the subject was able to control the position of a paddle in a computer game. Second, we examined whether the system is dependent on eye movements, since it is known that eye movements and auditory attention may influence each other, and any dependence on the ability to move one’s eyes would be a barrier to translation to paralyzed users. We discovered that, despite instructions, some subjects did make eye movements that were indicative of the direction of attention. However, there was no correlation, across subjects, between the reliability of the eye movement signal and the reliability of the BCI system, indicating that our system was configured to work independently of eye movement. Together, these findings are an encouraging step forward toward BCIs that provide practical communication and control options for the most severely paralyzed users.

## 1 Introduction

Brain-computer interfaces (BCI) are a topic of research and development that has seen increasing interest in the last 20–30 years (see Wolpaw et al., 2002; Wolpaw and Wolpaw, 2012, for review). The goal of BCI research is to develop systems that decode useful information from ongoing brain activity in real time. In most cases, that information is encoded voluntarily by the user (for example, by performing a voluntary mental operation to produce a measurable signal that can then be used for controlling some device, or by selectively attending to one of a set of stimuli in order to encode a choice). The result, according to the definition of Wolpaw and Wolpaw (2012), is a system that can replace, restore, enhance, supplement, or improve conventional central-nervous-system outputs. One of the most commonly considered goals is the development of communication systems for people who are *locked-in* by a paralyzing disease or accident (Bauer et al., 1979; Kübler and Birbaumer, 2008) – an example of *replacement* of an important function normally served by the motor system.

Within this field, there has been a recent increase in interest in BCI systems that are based on purely non-visual input. This is motivated by the desire to reach users in the most severely paralyzed states, for whom spatial vision may become extremely limited by the inability to open, direct, or focus the eyes voluntarily, by the inability to make saccades to integrate multiple fixations into a visual scene, and by the frequent infections that result from the lack of blinking (a review of some of these problems, in the particular case of amyotrophic lateral sclerosis, is provided by Sharma et al., 2011). Therefore, BCI systems that rely on visual stimuli may work less well that expected (Brunner et al., 2010; Treder and Blankertz, 2010), or not at all, for the users who need BCI most. Several recent approaches have presented multiple types of auditory stimuli, and required users to make a voluntary choice by covertly shifting their attention to one stimulus type while ignoring the others. This causes measurable modulation of auditory event-related potentials (ERPs; Hill et al., 2005; Sellers and Donchin, 2006; Furdea et al., 2009; Klobassa et al., 2009; Schreuder et al., 2009, 2010, 2011; Guo et al., 2010; Kanoh et al., 2010; Belitski et al., 2011; Halder, 2011; Höhne et al., 2011; Vlek et al., 2011; Hill and Schölkopf, 2012; Lopez-Gordo et al., 2012a,b) or steady-state evoked potentials (SSEPs; Kallenberg, 2006; Farquhar et al., 2008; Lopez et al., 2009; Kim et al., 2011). Auditory ERPs have been shown to provide a more reliable basis for BCI than auditory SSEPs (Hill and Schölkopf, 2012). Auditory BCI systems can also be divided according to whether they use a *streaming* or *sequential* technique. In streaming, streams of auditory stimuli are presented simultaneously or in rapid alternation, and the BCI system exploits the fact that the brain produces a different response to every stimulus in the attended stream when contrasted with every stimulus in the unattended stream (Hill et al., 2005; Kallenberg, 2006; Farquhar et al., 2008; Lopez et al., 2009; Kanoh et al., 2010; Kim et al., 2011; Vlek et al., 2011; Hill and Schölkopf, 2012; Lopez-Gordo et al., 2012a,b). In sequential presentation, relatively infrequent *target* stimuli are presented among more-frequent non-targets, and the BCI uses the difference in brain responses between targets and non-targets (Sellers and Donchin, 2006; Furdea et al., 2009; Klobassa et al., 2009; Schreuder et al., 2009, 2010, 2011; Guo et al., 2010; Belitski et al., 2011; Halder, 2011; Höhne et al., 2011). Streaming techniques are better suited for interfaces that provide a simple binary choice (such as a “yes” vs. “no” decision), whereas sequential techniques are better suited for higher-capacity systems that allow the user to select a letter (see Hill and Schölkopf, 2012, for more detailed discussion). Both approaches are useful as assistive communication tools: although a simple yes-no interface has a very limited range of expression relative to a speller, it is easier to learn because the user does not have to keep the assignment between stimuli and letters (or groups of letters) in memory. Therefore, it is a useful first step in establishing communication with severely impaired users, and may also be all that is needed to support some important practical tasks.

In this paper, we examine a two-class auditory streaming BCI system based on ERPs. This is very similar to that presented by Hill and Schölkopf (2012), and comparable to Hill et al. (2005), Kanoh et al. (2010), Vlek et al. (2011), Hill and Schölkopf (2012), and Lopez-Gordo et al. (2012a,b). Streams are presented dichotically, i.e., one to each ear, and each stream consists of a regular periodic sequence of tones repeated approximately twice per second. The effectiveness of auditory streaming BCIs in online usage has been established only recently (Hill and Schölkopf, 2012; Lopez-Gordo et al., 2012a,b) and, so far, only in healthy subject populations. In this study, our aim is to deepen our understanding of these systems and to improve their performance, making them more robust in advance of their application in clinical use. To this end, we will be asking two principal questions: first, which of the two variants that have been reported (the fixed-phase or the drifting-phase approach) is better? Second, do eye movements play a role in healthy subjects’ performance with the BCI, such that their level of performance may be unrepresentative of users who cannot move their eyes?

**Figure 1:**
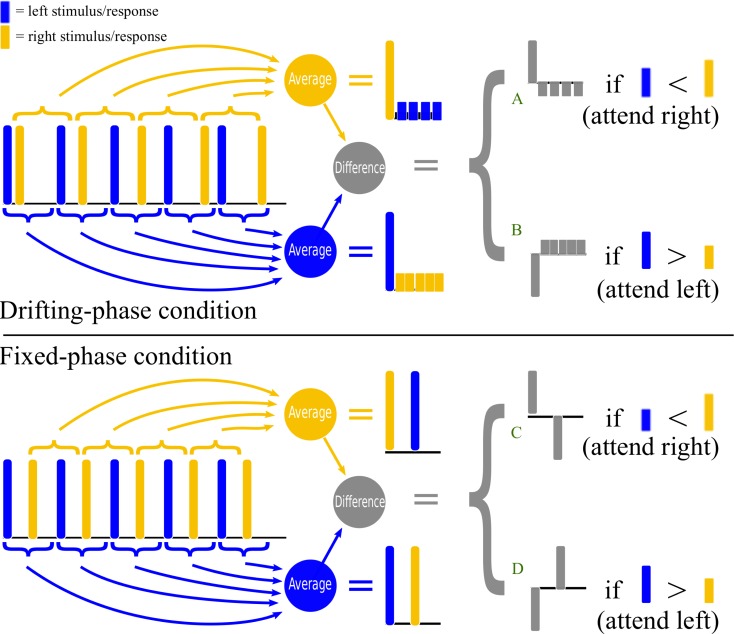
**The upper half of the figure shows a schematic illustration of stimulus timing and signal processing in the drifting-phase (DP) stimulus condition of Experiment I. The lower half provides a similar illustration for the fixed-phase (FP) stimulus condition of Experiments I and II**. Stimuli in the left stream (or the brain responses to them) are represented by dark blue pulses, whereas stimuli in the right stream (or the brain responses to them) are represented by light yellow pulses. Preprocessing consists of computing an average epoch time-locked to the left-stimuli onsets, and subtracting it from the average epoch time-locked to the right-stimuli onsets (see text for further details).

Our first aim is to compare two variations on the two-class auditory streaming approach, which we will call the drifting-phase (DP) and fixed-phase (FP) conditions. They are illustrated in Figure 1. In this figure, the times of the stimuli in the left channel are shown as dark blue bars and the stimuli in the right channel as lighter yellow bars. For clarity of illustration, we will also think of the bars as schematically representing the measured brain response to each stimulus (of course, in reality the EEG response is not a single instantaneous impulse, but the following considerations would apply equally well if we were to convolve these impulses with a more realistic event-related potential shape at a realistic latency).

The drifting-phase condition was used in the original auditory streaming design of Hill et al. (2005), and validated in the online study of Hill and Schölkopf (2012). In this approach, the left and right streams have unequal periods, so that, when we look at a series of epochs time-locked to the stimuli in one stream, the phase of the stimuli in the other stream appears to “drift.” This is illustrated in the upper half of Figure 1. Note that, if we compute an average response time-locked to the left-channel stimuli, the responses to the individual left-channel stimuli add together in phase, whereas the responses to the right-channel stimuli are spread out, and hence have a lower amplitude in the average. The converse situation is observed when averaging relative to the right-channel stimuli. The BCI control signal is based on the difference of these two time-locked averages. Therefore, if the right-channel stimuli are attended, and the responses to the right stimuli are consequently slightly larger than those to the left, the classifier sees a large initial response to the attended stimulus, followed by several negated responses to the unattended stimuli, attenuated, and at a number of different latencies (marked “A” in the figure). If the left-channel stimuli are attended, then the classifier sees a negative version of the same pattern (marked “B”).

By contrast, in the fixed-phase condition (lower half of Figure 1), the two streams have equal periods, with a constant anti-phase relationship. This means that whenever we compute an average response that is time-locked to the stimuli in one stream, the other stream’s stimuli will also be represented, delayed but at equal strength, in the same epoch. The classifier, looking at the difference between time-locked averages, must now discriminate between the patterns marked “C” and “D” in the figure. We might expect C-vs.-D to be an easier classification problem than A-vs.-B – not necessarily because of the larger amplitude of the delayed component (the information it carries will be very highly correlated with that carried by the initial component) but perhaps because of the smaller degree of overlap between the delayed and the initial components.

To confirm this preliminary intuition, we performed simulations assuming a simple one-channel biphasic response (a one-cycle wavelet with its negative peak at 100 ms and its positive peak at 300 ms). This was convolved with the impulse trains sketched in Figure 1 (actually parameterized more precisely in Section 2.3) assuming a 20% higher amplitude when a stimulus is attended than when unattended. The resulting waveform was added to 1000 different instances of pink noise whose standard deviation was twice that of the signal. This was then passed through our signal-processing chain (as described in Section 2.4) and the resulting preprocessed waveforms were assessed using a d-prime analysis. The results suggested that FP would indeed produce a signal-to-noise ratio up to 3 times higher than the DP in the features used for classification of attended vs. unattended trials.

A further attractive feature of the FP design is that it would allow information to be transferred more smoothly as a function of time (by contrast, in the DP design, stimuli from the two streams occur close together in time on some occasions, and further apart at others, so the instantaneous ability to discriminate attended from unattended streams varies over time). Since stimuli are presented frequently, roughly four times per second, and all stimuli are used by the classifier, the classifier output can be updated frequently. If this output were smoothed, it might therefore be possible for the FP design to mediate a form of *continuous* control, which has thus far not been demonstrated with ERP-driven brain-computer interface systems. In turn, such a system might be used to implement a new form of neurofeedback, as suggested by Hill and Schölkopf (2012), for potential use as a therapy in cases of attentional dysfunction, or for training in professions that put demands on one’s auditory stream segregation abilities, such as simultaneous interpreting or air-traffic control.

However, it is not clear whether a switch from DP to FP will actually result in improved BCI performance, since the evoked responses may be different in the two situations. Effective use of a streaming BCI requires that a human listener successfully focus attention on one stream and ignore the other, and this requires good perceptual segregation of the stimuli into separate auditory streams. Segregation of auditory streams is a complex phenomenon that is influenced by a wide range of physical stimulus properties that serve to “group” the stimuli in one stream together and distinguish that stream from other streams (Bregman, 1994). There is evidence that auditory attentional mechanisms lock on very readily to periodic stimuli (see for example Jones et al., 1981, 2002) and thus provide an attentional advantage to stimuli that are expected according to a regular schedule. Therefore, in the case where two streams have different (but constant) periods, we expect that stimulus period can serve as a strong cue by which stimuli can be grouped. Indeed, it is a striking subjective property of the DP stimuli that the unattended stream seems “easier to ignore” than in it is in the FP condition, where unattended stimuli occur predictably and exactly in the middle of the gap between attended stimuli.

The fixed-phase design was used by Kanoh et al. (2010) and Vlek et al. (2011) in offline studies and by Lopez-Gordo et al. (2012a,b) online. Drifting-phase was used by Hill et al. (2005) offline and Hill and Schölkopf (2012) online. Note that, despite the theoretical attractiveness of FP, it is the DP design of Hill and Schölkopf (2012) that has reported the best information transfer rate so far among auditory streaming studies. At the same time, several other factors, such as the number of EEG channels, stimulus rate or other stimulus characteristics, signal-processing methods, and individual difference between subjects, may account for differences between the studies that used FP and those that used DP. Consequently, the first aim of our study was to conduct Experiment I, which performs a direct within-subject comparison between these designs.

Our second aim was to investigate the role of eye movements. In all of our current and previously reported experiments, subjects were instructed to maintain fixation on a cross in the center of a screen. However, we have previously had no empirical confirmation that the subjects actually fixated. In fact, one of the subjects in Experiment I reported that he did not maintain fixation, and that he believed his changes in fixation made the attention task easier. This subjective report finds foundation in the literature since it is known that selective attention in a dichotic listening task tends to be associated with eye movements (Gopher, 1973) and even that eye movements may have a causal influence on one’s ability to direct auditory attention (Spence et al., 2000). In consequence, and in preparation for eventual application of auditory streaming BCIs to people with limitations in gaze, we performed Experiment II to determine to what extent performance on the auditory streaming task was dependent on eye movements that the target user population might be unable to produce.

## 2 Materials and Methods

### 2.1 Subjects

We conducted Experiments I and II using two non-overlapping groups of healthy subjects. There were 16 subjects in Experiment I (7 male: 9 female; 1 left-handed; mean age 32.6 ± 13 years) and 8 further subjects in Experiment II (5 male: 3 female; all right-handed; mean age 32.3 ± 13 years). Some of the subjects had had experience with BCI systems based on visual evoked potentials and sensorimotor rhythms, but none had practiced with this particular task.

One additional subject, who was experienced in auditory streaming BCI tasks, served as the subject in a pilot demonstration of continuous control.

All subjects had normal or corrected-to-normal vision and no history of significant hearing problems or neurological defects. Subjects gave informed consent according to the rules of the Institutional Review Board of New York State’s Department of Health.

### 2.2 Hardware and software

EEG recordings were made using a 16-channel g.USBamp series B amplifier (g.tec medical engineering GmbH, Austria) in conjunction with a 16-channel EEG cap (Electrocap Inc.). The cap used gelled 9 mm tin electrodes at positions F3, Fz, F4, T7, C3, Cz, C4, T8, CP3, CP4, P3, Pz, P4, PO7, PO8, and Oz of the extended international 10–20 system of Sharbrough et al. (1991), with the reference at TP10 (the right mastoid) and the ground electrode at TP9 (the left mastoid). The amplifier performed appropriate anti-alias filtering before digitizing at 24 bits and downsampling to 256 Hz. A second g.USBamp was synchronized to the first, and provided two auxiliary channels for precise recording of auditory stimulus timing. Data acquisition was performed using the BCI2000 software platform (Schalk et al., 2004; Schalk and Mellinger, 2010) v.3.0; signal-processing and stimulus presentation was implemented in Python using the “BCPy2000” add-on to BCI2000 (Hill et al., 2007). Classification was performed using Matlab (The MathWorks, Inc). The software ran on an Lenovo ThinkPad T61p laptop with a 2.2 GHz dual-core processor. Stimuli were delivered via the laptop’s built-in soundcard, simultaneously to a pair of Sony MDR-V600 headphones worn by the subject and to the auxiliary amplifier channels (the filtering and downsampling to 256 Hz naturally lost much of the sound signal content, but this system provided a sufficiently precise record of the stimulus onset times). Visual cues were presented at a comfortable distance (roughly 60 cm) using an LCD flat-screen monitor with a 60 Hz refresh rate. In Experiment II, this was a Tobii T60 eye-tracking monitor (Tobii Technology, Sweden).

### 2.3 Stimuli and task design

One *trial*, i.e., one cued attempt to attend consistently either to the left or to the right, consisted of 8 stimuli in the left-stream interleaved with 7 stimuli in the right stream. Each stimulus was a sawtooth wave lasting 150 ms, including 5 ms rise and 5 ms fall times during which a raised-cosine envelope was used. Stimuli were anti-aliased for digitization and synthesis at 44.1 kHz. Left-stream stimuli were delivered only to the left earphone and had a fundamental frequency of 512 Hz, whereas right-stream stimuli were delivered only to the right earphone and had a fundamental frequency of 768 Hz.

In the left stream, stimuli were delivered with a fixed-stimulus onset asynchrony (SOA) of 492.2 ms. In the fixed-phase (FP) condition, this same SOA was also used for the right stream, but the stream started half a period later (i.e., the SOA between the first left-stream stimulus and the first right-stream stimulus was 246.1 ms). This led to a stimulus pattern similar to the one sketched in the lower half of Figure 1. In the drifting-phase (DP) condition, the SOA within the right stream was 546.9 ms and the initial SOA between left and right was 109.4 ms (see the upper half of Figure 1).

Each stimulus could be either a *standard* or a *target* stimulus. Standard stimuli sounded “rough” because they were amplitude-modulated to 100% depth by a raised sinusoidal envelope at 42.67 Hz (left) or 36.57 Hz (right), corresponding to modulation periods of exactly 6 or exactly 7 EEG samples. In contrast, target stimuli sounded “smooth” because they were not amplitude-modulated. Within one trial, the first two stimuli in each stream were always standards; then, of the remaining stimuli, there could be 1, 2, or 3 targets, which subjects were required to count. The number of targets was chosen randomly, uniformly, and independently for each stream on each trial.

Thus, the design of the stimuli and trials was very similar to that of Hill and Schölkopf (2012) with three minor differences. First, there were very slight differences in the numerical values of the offsets and frequencies, due to different hardware constraints. Second, targets were distinguishable from standards by the amount of amplitude-modulation rather than by duration (we considered it desirable to have equal-duration tones throughout, in order to make the timing of the brain responses more uniform). Third, there was no “background” sound between stimuli, a design feature that was only useful for the very specific purposes of Hill and Schölkopf (2012).

At the start of each trial, subjects were given a visual cue (either the text <<< *LEFT* or the text *RIGHT* >>> presented in the center of the screen for 2 s) instructing them which stream to attend to. Half the trials in a given run were left, and half were right, in random order. Subjects were instructed that from the moment the cue appeared, they should keep their gaze fixed on the center of the screen, and refrain as much as possible from blinking, swallowing, or moving. The cue was displayed for 2 s before being replaced by a fixation cross, and the sound stimuli began after an additional 500 ms. The fixation cross remained for 5 s (by which time the stimuli had been finished for at least 500 ms) and was then replaced by a question-mark. This signaled to the subject that they were free to blink, swallow, or move. At this moment, they received acoustic feedback (a single “ding!” of a bell) if the system had correctly classified attention-to-the-left versus attention-to-the-right using the EEG. The question-mark also signaled that the subject had up to 5 s in which to press a key on their numeric keypad to report how many target stimuli had been in the attended stream. As soon as they pressed the key (or after 5 s had elapsed), the screen displayed, for 2 s, the correct number of targets in each stream: the numeral on the attended side was green if the subject had responded correctly, red if not. After a pause of 1–2 s, the next trial began.

Trials were performed in runs of 20, a run lasting about 3.5 min, after which the subject could take a break for a minute or two. Before the first run, subjects were asked to listen to the stimulus a few times, practicing the counting task and, between repetitions, adjusting the volume of the left and right headphone outputs using two analog sliders. The criteria were that the volume should be comfortable, and that attending to the left stream and ignoring the right should be, subjectively, equally easy as vice versa. Each subject then performed 12 runs over the course of about an hour. The stimulus condition was switched every three runs (this was a compromise: we wished to interleave the DP and FP conditions in order to compare them on as equal a footing as possible, but we did not wish to confuse the subjects by changing the stimuli too often). Half the subjects (randomly chosen) performed the runs in the order FP1-FP2-FP3, DP1-DP2-DP3, FP4-FP5-FP6, and DP4-DP5-DP6, whereas the other half performed them in the order DP1-DP2-DP3, FP1-FP2-FP3, DP4-DP5-DP6, and FP4-FP5-FP6. Runs FP1 and DP1 were performed without the feedback bell, since classifier weights had not yet been computed for the stimulus condition in question. Thereafter, the classifier was re-trained and updated at the end of each run, using as training data all the runs gathered so far in the relevant stimulus condition. Therefore, in each stimulus condition there were 5 runs (or 100 trials) during which online performance could be assessed.

#### 2.3.1 Continuous control pilot

In the continuous control demonstration, only the FP condition was used. The stimuli were identical to those of Experiment I except that, after three initial 20-trial runs for calibration, the experiment no longer used a trial structure and did not display explicit instructions. Instead, the auditory stimuli alternated continuously left and right from the beginning of the run until the moment the run was manually stopped, and the subject could freely shift attention back and forth between streams. The BCI’s classifier’s un-thresholded output was used as a control signal in a computer game, specifying the horizontal velocity of the bat in a version of the classic “Breakout” (Bushnell et al., 1976) presented on screen. The player’s task was to guide the bat left or right so as to keep a ball bouncing against a disintegrating wall of bricks. The only major difference between this and conventional Breakout was that there were no “lives”: whenever the ball went off-screen, it simply re-appeared with the same velocity and position it had had 1 s before, and descended again along the same path.

#### 2.3.2 Experiment II differences

The stimuli of Experiment II were almost identical to those described above except that the within-stream SOA was exactly 500 ms and the SOA between streams was exactly 250 ms. Also, the DP condition was not used. The FP condition was instead interleaved, by the same method of alternating every three runs, with a third stimulus condition that will not be reported here – only the eye-tracking results in the FP condition will be considered.

### 2.4 Online signal-processing and classification

Online signal-processing methods and classification methods were identical to those of Hill and Schölkopf (2012), and are described and discussed in more detail in that paper. To summarize: the 16-channel EEG signal was band-pass filtered between 0.1 and 8 Hz using a Butterworth filter of order 6. The first two stimuli of each stream were disregarded, after which overlapping epochs of duration 600 ms were extracted, starting at the onset of every stimulus. The running average of epochs time-locked to the left-stream stimuli, *X_L_*, was subtracted from the running average of epochs time-locked to the right-stream stimuli, *X_R_*, to yield a feature set *X*_Δ_ for classification. Classifier weights were computed using a linear logistic regression classifier, L2-regularized in a transformed space – specifically, the space of signals after symmetric whitening, i.e., premultiplication by the inverse matrix square root of the 16 × 16 spatial covariance matrix averaged across all training epochs. (We demonstrate and explore the importance of the whitening step in Farquhar and Hill, 2013.) The BCI output signal, computed as the sum of the feature values weighted by these classifier weights, was updated every time a new epoch became available (roughly 4 times per second) and the sign of its final value was used as the prediction for each trial.

In the continuous control demo, we used an exponential moving average with a decay factor of 0.5. The un-thresholded output signal was further smoothed by filtering with an 8th order Butterworth lowpass filter with a cutoff at 3 Hz. This signal was then associated with the horizontal velocity of the game paddle.

## 3 Results

### 3.1 Experiment I: Fixed-phase vs. drifting-phase

**Figure 2:**
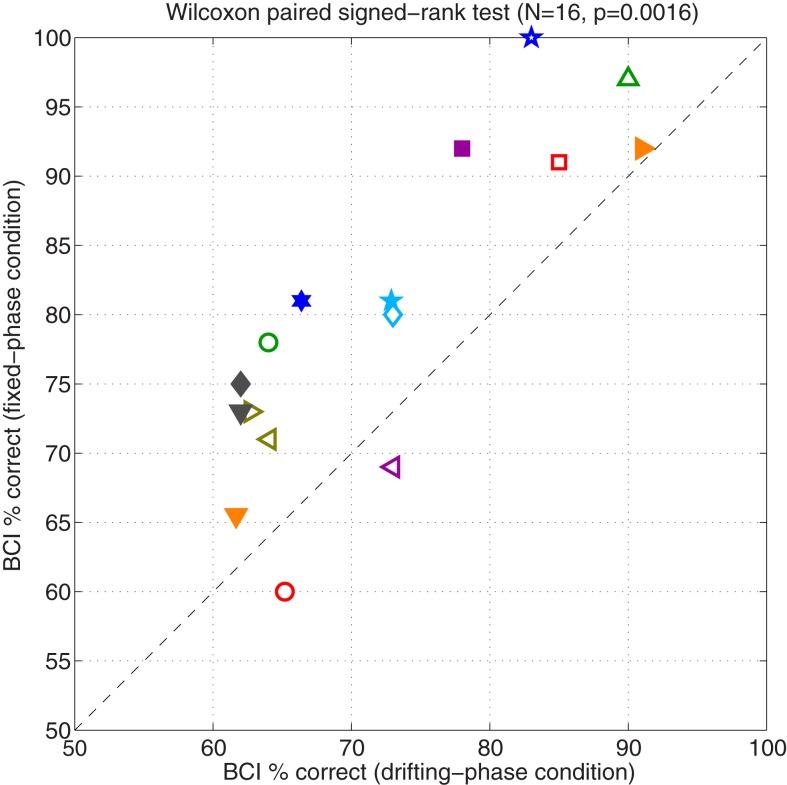
**This figure shows the main results of Experiment I**. Each symbol represents data for a particular subject. Performance on the fixed-phase condition is plotted against performance on the drifting-phase condition. Performance is calculated as the average accuracy of the classifier, calculated across the 100 online trials available in each condition. Accuracy due to chance was 50%. Open symbols denote subjects who started with the DP condition; filled symbols denote those who started with FP.

In each stimulus condition of Experiment I, there were 100 trials in which online feedback was given. Figure 2 shows the percentage of these trials that were classified correctly online by the BCI system. Performance in the FP condition is plotted against performance in the DP condition, for each subject. Chance performance was 50% in either case. Open symbols denote subjects who did the DP condition first, and filled symbols denote those who started with the FP condition. The FP condition was superior for 14 out of the 16 subjects: online binary BCI classification performance ranged from 60/100 to 100/100 correct across subjects (mean: 79.9%, standard deviation: 11.7%), an increase of 7.8 percentage points relative to the DP condition (mean: 72.1% ± 10.4, range: 62–91). A non-parametric paired test showed that the increased performance of the FP condition was statistically significant (Wilcoxon signed rank test: *N* = 16, *p* = 0.002).

To characterize the EEG responses that drive this BCI system, we performed two analyses of the data from the fixed-phase condition. First, we computed a grand-average response to all the (left-attended, right-unattended) stimulus pairs, and another grand average to all the (left-unattended, right-attended) stimulus pairs. Second, since a grand average does not necessarily highlight the sources that have the greatest signal-to-noise ratio (which are therefore most informative for single-trial discrimination), we computed each feature’s sensitivity index *d_a_* (Simpson and Fitter, 1973) as follows:

$$d_{a}=\frac{\left(\mu_{\text{attended}}-\mu_{\text{unattended}}\right)}{\sqrt{\left(\sigma_{\text{attended}}^{2}+\sigma_{\text{unattended}}^{2}\right)}/2}$$

**Figure 3:**
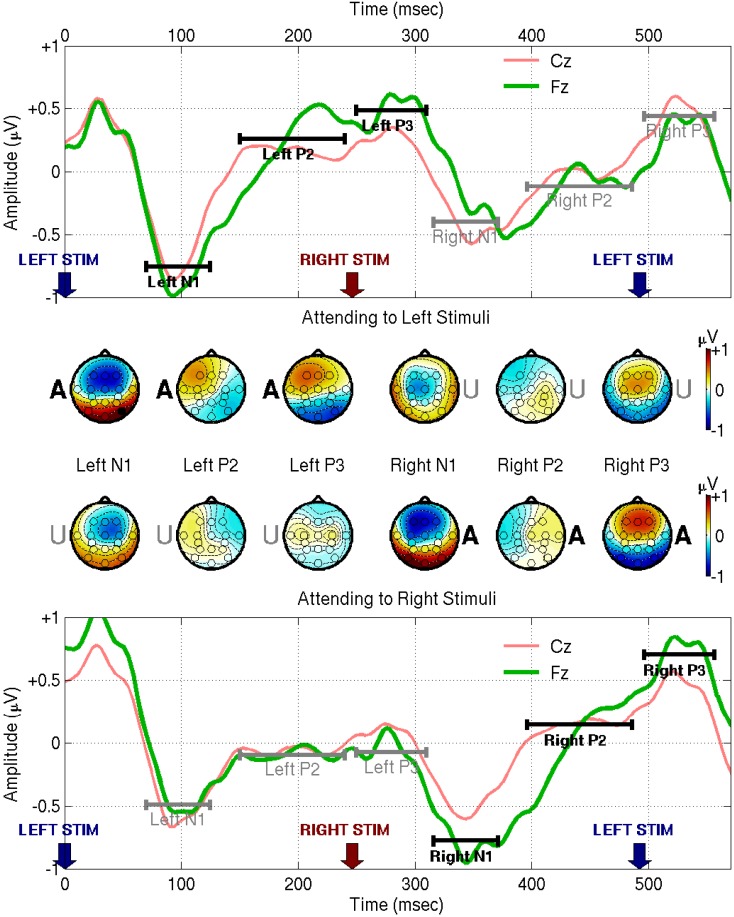
**This figure shows EEG responses from the fixed-phase condition of Experiment I, averaged across all subjects**. For visualization purposes the signals were band-pass filtered between 0.1 and 45 Hz using a zero phase-distortion method, and re-referenced to the common-average. The upper half of the figure shows responses to an attended left-ear stimulus at time 0 followed by an unattended right-ear stimulus at time 246.1 ms. The lower half of the figure shows the converse case: an unattended left stimulus followed by an attended right stimulus. The range 70–125 ms following each stimulus presentation is marked as N1, 150–240 ms is marked as P2, and 250–310 ms is marked as P3 (these numerical values were chosen based on the traces of Cz and Fz themselves, taking account of both electrodes, and both left- and right-attended stimuli). Scalp topographies were computed by averaging the signal within each of these ranges. The letters A and U appear at one or the other ear position on the scalp maps, to denote the ear in which the eliciting stimulus was delivered and whether it was [A]ttended or [U]nattended.

The grand-average results are shown in Figure 3. The first thing to note is that an attended stimulus produces a higher-amplitude cycle of ERP waves at Cz and Fz than an unattended stimulus – this is the phenomenon on which the BCI system relies. There appear to be three components of interest, so we define three temporal ranges of interest based on the shape of the Cz and Fz traces following attended stimuli. We label these ranges as N1 (70–125 ms), P2 (150–240 ms), and P3 (250–310 ms), and show the corresponding scalp topographies obtained by averaging within each range. The responses to unattended stimuli, depicted in the scalp maps that have a letter U at the position of the stimulated ear, show a fronto-central N1 profile with a distinct contralateral shift, as also reported by Woods and Clayworth (1987). By contrast, the N1 response to attended stimuli (denoted by an A at the stimulated ear) is more symmetric about the midline. The relatively weak P2 component manifests ipsilaterally, and shifts in a frontal direction when the stimulus is attended. The P3 component, relatively weak for unattended stimuli, is prominent in frontal locations following attended stimuli, with a possible ipsilateral shift that is only obvious for left-ear stimuli.

**Figure 4:**
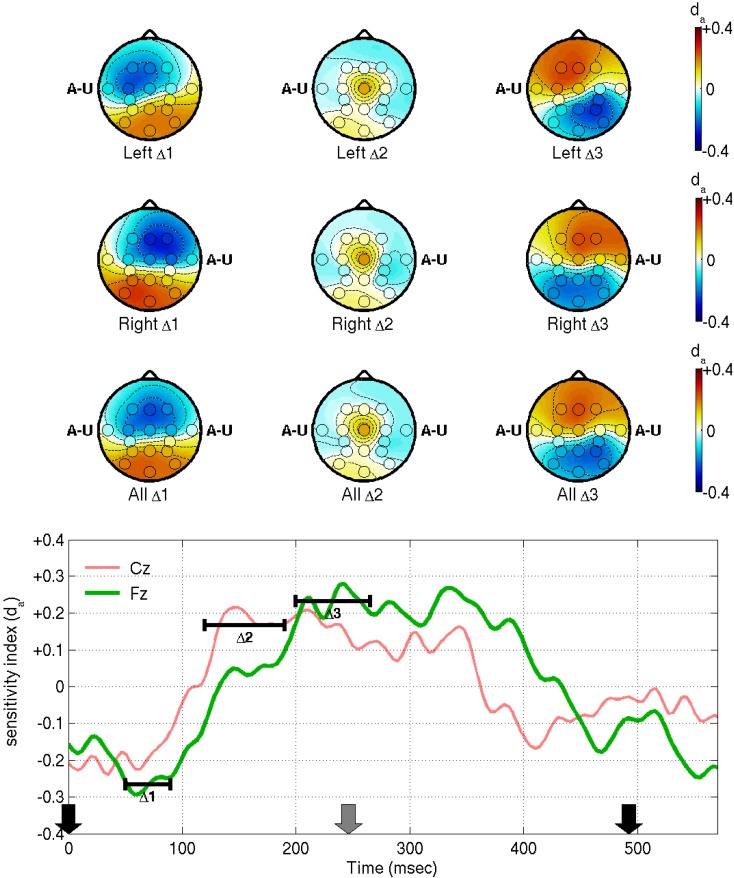
**This figure shows the reliability of EEG features in distinguishing attended from unattended stimuli in the fixed-phase condition of Experiment I, as measured by the sensitivity index, *d_a_***. Before computation of *d_a_*, the features were processed as in Figure 3, i.e., band-pass filtered between 0.1 and 45 Hz, referenced to the common-average and averaged within each trial. The *d_a_* scores were then computed and averaged across all subjects. The bottom panel shows *d_a_* for electrodes Cz and Fz as a function of time, relative to the stimulus onset times marked by the arrows (black and gray arrows denote stimuli from opposing streams). The ranges marked Δ1, Δ2, and Δ3 were chosen based on these traces, at 50–90, 120–190, and 200–265 ms, respectively. Scalp maps show *d_a_* averaged within each of these ranges, for left-ear stimuli at time 0 (first row), right-ear stimuli at time 0 (second row), or for all stimuli irrespective of laterality (third row). The letters A–U appear at the ear positions of the scalp maps, as a reminder of the ear stimulated at time 0 and of the fact that, unlike those of Figure 3, the topographies represent a contrast between attended and unattended stimuli.

The contralateral N1 to unattended stimuli and more-symmetric N1 to attended stimuli mean that the difference wave appears to manifest ipsilaterally, as we see in the first two rows of Figure 4. This is at odds with the symmetrical “early Nd” distributions reported by Woods and Clayworth (1987): we cannot offer a full explanation of this discrepancy, but note that there are many differences between the two studies’ stimulus designs, most notably the fact that our stimuli are strictly periodic, whereas the earlier authors used randomized inter-stimulus intervals. Note that the early negative difference wave peaks somewhat earlier than the N1 itself, at 50–90 ms, marked as Δ1 in Figure 4. This range and the other ranges of interest, Δ2 (150–240 ms) and Δ3 (250–310 ms), were again chosen based on the empirical Cz and Fz traces. Note that, looking at the difference wave alone, it is hard to distinguish where modulation of the late positive peak ends and modulation of the next early negative peak begins: with an attended stimulus at time 0, potentiation-by-attention of the first P3, and attenuation-by-inattention of the subsequent N1 both entail a shift in the direction of positive voltage. Likewise, when the stimulus at time 0 is unattended, attenuation-by-inattention of the P3 and potentiation-by-attention of the subsequent N1 both entail a negative voltage shift. Therefore, range-of-interest Δ3 was cut short to minimize confusion by contributions from ERPs elicited by the subsequent stimulus. Generally, however, the positive peaks of the difference waves align well in time with the corresponding positive peaks in the grand average. The Δ2 difference has a markedly higher signal-to-noise ratio at Cz than anywhere else. Finally, the positive Δ3 difference is located frontally, consistent with Woods and Clayworth’s “late Nd” component, although again we find an ipsilateral shift where the previous authors did not.

The third row of scalp plots, and the traces in the bottom panel of Figure 4 show *d_a_* for attended vs. unattended stimuli disregarding laterality. This is of interest because it is equal to $1/\sqrt{2}$ times the *d_a_* of the right-left difference features *X*_Δ_ that are used directly for classification of attend-left versus attend-right trials. Hence, it shows the locations that are likely to be most relevant for our classifier. Overall, the latencies and the frontal bias are broadly consistent with the early observations of Hansen and Hillyard (1980) and Woods and Clayworth (1987) on endogenous early and late Nd components (otherwise referred to as Nd and P3, respectively, in the report of Woods and Courchesne, 1986). The results are also consistent with the scalp maps that have previously been estimated for this BCI design, as provided in higher resolution than the current montage allows in Figure 5 of Hill and Schölkopf (2012).

There are similarities and differences between our response profiles and those shown by other auditory BCI systems. An early negative component followed by two positive peaks, similar to our Figure 3, is also characteristic of the target responses shown by Schreuder et al. (2011) in their Figure 6. A further point of agreement is that Schreuder’s early negative component, when averaged across all spatial stimulus locations, has a frontal midline spatial pattern. Based on latency, however, they identify this component as N2 rather than N1. Guo et al. (2010) also identify a useful N2 response, this time with a more central scalp location. The P3 component identified by Schreuder and by Guo has a longer latency than ours and a more-posterior location, peaking at the C and CP electrodes in Schreuder’s study, and at the P and PO electrodes in Guo’s. The longer latencies and the more-posterior tendency of these authors’ ERP components may be related to the difference in paradigm, since both studies used a target-ERP rather than a streaming approach, and hence much longer intervals between attended stimuli.

### 3.2 Pilot demonstration: Continuous control in the fixed-phase condition

After three calibration runs of 20 trials each, the subject practiced with the Breakout game for a total of 31 min. The first 15 min were broken up into 7 unequal runs that were used to optimize parameters of the BCI system, such as the gain and offset of the control signal. The final 16-min game play can be seen in Movie S1 in Supplementary Material.

**Figure 5:**
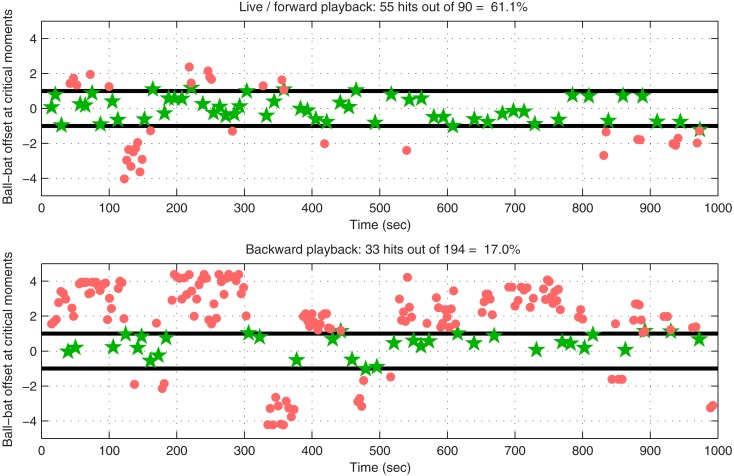
**Performance during a 16-min run in which one subject played our modified Breakout game by controlling the velocity of the paddle using shifts of selective attention between streams of stimuli in the two ears**. The horizontal axis represents time in seconds, and the vertical axis represents horizontal distance between the center of the bat and the center of the ball, with ±1 indicating the ends of the bat itself. Green stars indicate hits (ball bounced upwards off the paddle), whereas red dots indicate misses (the ball fell past the paddle, very soon falling off-screen and “re-spawning” to repeat the last second of its downward trajectory). The upper panel shows performance during forward playback of the EEG recorded during the game (results are identical to those obtained by analyzing bat and ball movements in the actual original game file), whereas the lower panel provides an estimate of chance performance by illustrating game performance when data are played backward.

While these are only exploratory results, and while a visually presented computer game is clearly not the main target application for an auditory BCI system, we were interested in quantifying performance, and in comparing it against an estimate of the performance we might expect under the null-hypothesis of no control. In this way we wished to obtain a preliminary idea of the feasibility of continuous control via auditory BCI. The upper panel of Figure 5 shows an analysis of performance during the final 16-min game: the horizontal distance between the center of the bat and the center of the ball is plotted as a function of time at those time points at which the ball either hit the bat (green stars) or dropped past it (red dots). The distance is normalized such that the width of the bat itself is 2 units: thus, an absolute bat-ball distance ≤1 indicates a solid hit, whereas an absolute distance >1 at the critical moment usually indicates a miss (given that the ball had non-zero radius, hits were still possible at distances slightly greater than 1, depending on the angle of incidence). In 16 min, the bat was successfully guided to bounce the ball upward 55 times, and it missed 35 times (a hit rate of 61.1%); the ball struck bricks 54 times, clearing the level (the game started with 18 red bricks each requiring two hits to clear, and 18 blue bricks each requiring a single hit).

To illustrate that the level of control achieved by this subject was better than chance, we first played back the recorded EEG signal and fed it through the same signal-processing and application pipeline as those used during the online game. The bat and ball positions recorded as a function of time in this playback run were identical to those in the original file, which verified the validity of the playback pipeline for this setup. The procedure was then repeated, but with the EEG played *backward*, thereby destroying any meaningful relationship between the bat’s movement and the movement that would be necessary to catch the ball. The results are plotted in the lower part of Figure 5: the number of hits decreased from 55 to 33, and the number of misses increased from 35 to 161, resulting in a “chance” hit rate of 17.0% as compared with the original 61.1%. Therefore, the control in the original game was better than chance (*p* ≪ 0.001 using Fisher’s exact test), an impression that is unmistakable when watching the video at high speed: through no more overt means than the act of listening, our subject was clearly able to play Breakout.

### 3.3 Experiment II: Eye position and movements in the fixed-phase condition

The online BCI performance of the subjects in Experiment II, with a mean of 82.1% correct and a standard deviation across subjects of 11.4%, was similar to the performance of the subject group in Experiment I. The main goal of Experiment II was to investigate the role of eye movements in explaining performance variation across individuals.

**Figure 6:**
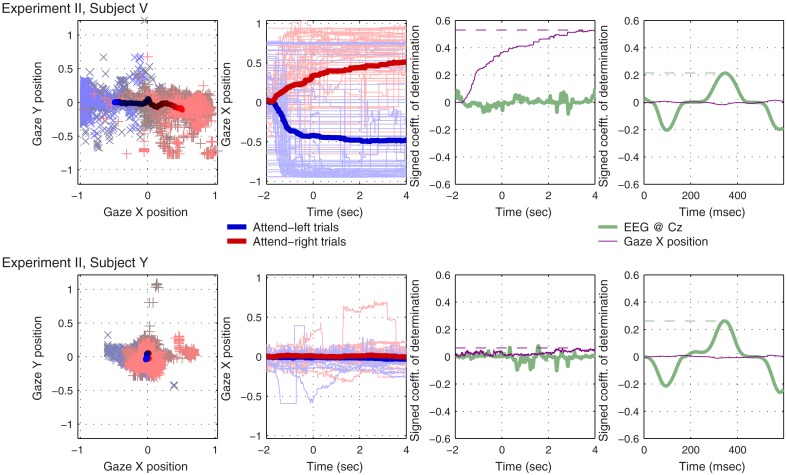
**Results from two example subjects that exhibited contrasting eye-gaze behavior during the 6 s following the presentation of the directional visual cue**. The first graph in each row shows gaze position in two-dimensional coordinates: individual trials are plotted as + and × in the background; means across trials are plotted as solid lines in the foreground; gray/black denotes the beginning of each trial, shading toward red (for attend-right trials) or blue (attend-left trials) over the course of 4 s. The second graph in each row shows just horizontal gaze position as a function of time: the directional cue was presented at *t* = − 2 s, and the sound stimuli started at *t* = 0. Again, attend-right trials are plotted in red, attend-left trials in blue, and the mean-across-trials, within each class, is plotted as a thick line. Individual trials are plotted as thin, paler lines. In the third graph, we see a measure of single-trial separability, the signed coefficient-of-determination (signed *r*^2^) as a function of time across the whole trial, for both the EEG signal at Cz (green) and horizontal gaze position (purple). Finally, in the fourth graph, we see signed *r*^2^ values for both Cz and horizontal gaze position, this time after preprocessing as described in Section 2.4.

**Figure 7:**
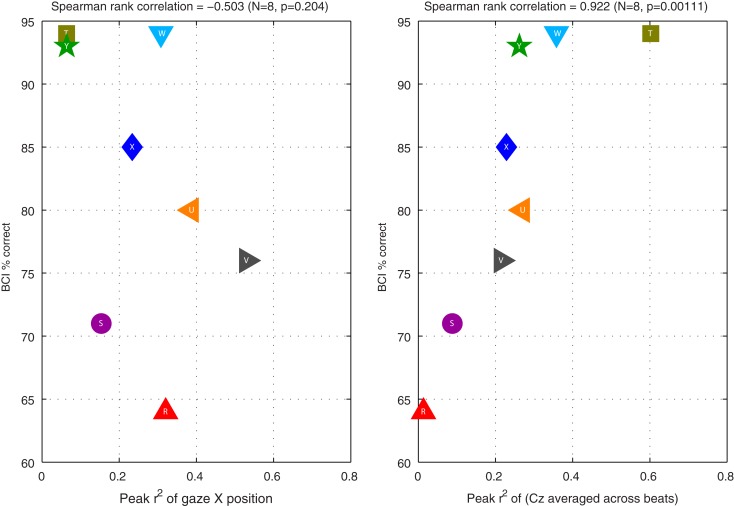
**Relationships between BCI performance (vertical axis) and *r*^2^ of certain features (horizontal axis) in Experiment II**. The left graph shows performance as a function of the peak *r*^2^ of uncut horizontal gaze position over the course of a trial (corresponding to the purple horizontal dashed lines in the third column of subplots in Figure 6). The right graph shows performance as a function of the peak *r*^2^ of the preprocessed EEG at Cz (corresponding to the green horizontal dashed lines in the fourth column of graphs in Figure 6).

Figure 6 shows two example subjects from Experiment II, subjects V and Y, who exhibit contrasting eye-gaze behavior. For each subject, the first graph shows two-dimensional gaze position. The second graph shows horizontal gaze position as a function of time since presentation of the visual “LEFT” or “RIGHT” cue. The third and fourth graph show a measure of separability of the individual attend-right trials from attend-left trials according to this horizontal gaze measure, as compared with the EEG signal at Cz. The third graph does so using the raw data, across time during the whole trial, and captures very well the predictive effect of static gaze position: the dashed horizontal purple lines mark the peak *r*^2^ values for horizontal gaze position, which will be used to characterize the whole subject group in Figure 7. The fourth graph is similar to the third, but this time the signals (both EEG and gaze parameters) have been band-pass filtered, averaged and subtracted in the manner employed by the BCI classifier (described in Section 2.4 and schematically depicted in Figure 1). The motivation for this last analysis was to investigate whether rhythmic eye movements (in time with the attended beats) might play a role in classifying the direction of attention, just as rhythmic EEG responses at Cz clearly do. (To provide a frame of reference for the eye-gaze *r*^2^ values, we will also compute peak values of *r*^2^ for preprocessed EEG at Cz, indicated by the green horizontal dashed lines, for use in Figure 7.)

Note that, despite instructions to maintain fixation, subject V reliably looks to the right when attending to the right, and to the left when attending to the left, to such an extent that the individual trials can be well separated by this measure. Subject Y, by contrast, maintains central fixation quite faithfully, and the individual trials cannot be classified according to horizontal gaze position. These two subjects illustrate two extremes of behavior in the subject group: in fact, each of the 8 subjects exhibited a characteristic gaze pattern in which gaze tended more or less toward the direction of attention, depending on the individual. Rhythmic eye movements did not seem to play a role: the very small *r*^2^ values that we see in the purple traces of the rightmost graphs for subjects V and Y are typical of the group: across all 8 subjects, the preprocessed gaze parameters never generated a peak *r*^2^ value more than 0.064, and the mean and standard deviation across subjects were 0.038  ± 0.014. We conclude that rhythmic eye movements played very little role compared with either static horizontal gaze position (*r*^2^ = 0.258 ± 0.162 across subjects) or preprocessed EEG (*r*^2^ = 0.255 ± 0.177).

It is interesting to note how similar the distributions of these latter two statistics are: horizontal gaze position, as single feature, is just as good a predictor of the direction of our subjects’ attention as the pre-processed EEG from a single channel. However, the important question is: does our BCI system inadvertently use this gaze information, thereby solving the BCI problem by means that a person who has limited control over eye movements could not replicate? Figure 7 addresses this by plotting BCI performance as a function of the two statistics in question. In the right panel, we clearly see that peak *r*^2^ of the preprocessed EEG is closely related to online BCI performance (Spearman rank correlation coefficient = 0.922; *N* = 8, *p* = 0.001). This is expected, because the BCI classifier is based on precisely this pre-processed feature (together with features obtained in the same way from other EEG channels). By contrast, in the left panel, we see that peak *r*^2^ of horizontal gaze position is not related to BCI performance (Spearman rank correlation coefficient =  −0.503; *N* = 8, *p* = 0.20). That is to say: if the system had been built to measure gaze position and factor it into the prediction of subjects’ direction of attention, then some of our subjects’ performance would undoubtedly have been boosted. However, the way the system is constructed, shifting gaze does not appear to help: those subjects who shifted their gaze more did not tend to be the subjects for whom the BCI worked better – if anything, the direction of the (non-significant) correlation even suggests the contrary. This is an encouraging indication that the system is constructed such that it does not rely on signals that could likely not be produced by the target users.

## 4 Discussion

In Experiment I, we determined that a fixed-phase (FP) alternation between stimuli is significantly more effective than a drifting-phase (DP) stimulus design, over the short fixed-length trials used here, for a large majority of subjects. This result seemed plausible *a priori* according to the logic of Figure 1, but was not certain, because the DP condition’s difference in periods between the two stimulus streams is a cue for auditory segregation that may assist the subjects in shifting their attention.

The considerable performance difference between the FP and DP conditions highlights the fact that, in general, the optimization of stimulus parameters in this relatively new BCI approach remains to be explored and may yield considerable improvements. One parameter of particular interest will be the speed of stimulus presentation. The strategy of Lopez-Gordo et al. (2012a) was to increase the left-right alternation rate to 5 Hz – more than twice the rate employed in the current study – in order to resonate with and drive the observed alternation between the N1 and P2 ERP components, 100 ms apart. This is an appealing idea, although it is far from clear, *a priori*, that it is necessarily optimal. The results of Hill and Schölkopf (2012) suggest a separation of 200 ms rather than 100 ms between the relevant negative and positive components. This is more in line with the expectation that, rather than the exogenous N1 and P2 components, it is the endogenous components Nd and P3 (225 ms apart in Woods and Courchesne, 1986), that are important when making inferences about attention. This would suggest a driving frequency closer to 2.5 Hz rather than 5 Hz. Nonetheless, theoretical optimality according to this or any other single criterion does not necessarily mean optimal performance, since speed can be expected to have many conflicting implications for such BCI systems. First, within-stream stimulus rate is known to have a large influence on the ability to segregate auditory streams (see Bregman, 1994, for review). Second, more-frequent stimuli naturally entail a higher theoretical limit on the information transfer rate in any ERP-based BCI system, assuming equal brain responses at all stimulation frequencies. Third, however, all brain responses *cannot* be assumed to be equal: too-rapid stimulation may cause refractory effects that attenuate the ERP components in question (Woods and Courchesne, 1986) – although attention- and task-dependent components may have a relatively short refractory period (Woods and Knight, 1986) and refractory effects may interact with the effects of habituation and training (Brattico et al., 2003). The trade-offs between these factors, and the resulting optimum stimulation rate, are not easy to predict in advance and will require careful comparative assessments.

It is now possible to move forward with the FP design with increased confidence, and this simplifies many possible extensions of the auditory streaming BCI paradigm. Since the opposing streams are no longer drifting in and out of phase with each other, information may be transferred more uniformly as a function of time. For two-class classification, this makes it easier to manage trials of variable length, such that the classifier has the option of waiting until a certain confidence threshold is exceeded – perhaps using some unsupervised method for assessing signal-to-noise ratio, such as that demonstrated by Lopez-Gordo et al. (2012b). Among BCI systems driven by event-related potentials, this property of uniformity is unique to streaming designs: by contrast, a sequential BCI system (for example, one specifically designed to harness the P300 response) only gains information from responses to relatively infrequent target stimuli – the classifier must wait for these targets to occur, at irregular intervals, before it can supply deliver new information. This makes streaming designs potentially suitable for a range of unique applications, such as real-time attention monitoring, rapid assessment of attentional skills, and neurofeedback training of attention. We have demonstrated, for the first time, that it may even be possible to exploit attention shifts to auditory streams to provide a continuous voluntary control signal: our trained subject was able to control a continuous system in response to complex task demands (those of the computer game Breakout) in real time.

Following the finding of Experiment I, Experiment II focused entirely on the fixed-phase design. Although the subjects were instructed to maintain fixation throughout each trial, Experiment II revealed that some achieved this better than others. Each subject exhibited a characteristic pattern of gaze behavior over time. When processed using the same pipeline used for EEG (i.e., when the signal is band-pass filtered and a between streams difference of time-locked averages to the individual stimulus beats is computed) eye-gaze parameters did not reflect whether the subject was paying attention-to-the-left or attention-to-the-right. It therefore seems unlikely that rhythmic eye movements, in time with the attended stimuli, played any role in BCI performance. Some subjects exhibited a reliable drift of gaze, over several seconds, in the attended direction. However, across subjects the reliability of this effect (measure by the peak coefficient-of-determination of the horizontal eye position signal over the course of the trial) did not correlate with the subjects’ performance in the BCI task, whereas (for example) the peak reliability of the preprocessed EEG at Cz does explain a large proportion of the cross-subject performance variability. Clearly, when using healthy subjects to test a system designed for paralyzed people, one must be cautious in interpreting the role of unrepresentative movements, such as controlled eye movements, that the healthy subjects may make – whether these are causal or epiphenomenal to the shifting of auditory attention. Nonetheless, we found no evidence that eye movement is necessary to, or even beneficial to, BCI performance in an auditory streaming paradigm. A final confirmation for this proposition requires the testing of a streaming-based BCI by the users for whom it is intended, i.e., people who cannot make controlled eye movements.

In summary, Experiment II gives us confidence that the promising performance levels achievable by auditory streaming BCIs are not explained by eye movements that the paralyzed target population would be unable to make. Experiment I established that, of the two published stimulus designs for auditory streaming BCI, the fixed-phase approach is more promising for further development – and the large magnitude of the difference highlights the potential benefits that might be achieved by further optimizing the stimulus parameters of auditory streaming BCIs. The findings bring auditory streaming BCI two steps closer to practical reality as a communication method for severely paralyzed people. Furthermore, we were able to demonstrate for the first time that the method might feasibly be harnessed for continuous control and neurofeedback applications.

## Conflict of Interest Statement

The authors declare that the research was conducted in the absence of any commercial or financial relationships that could be construed as a potential conflict of interest.

## Supplementary Material

The Supplementary Material for this article can be found online at http://www.frontiersin.org/Neuroprosthetics/10.3389/fnins.2012.00181/abstract
